# 2020 SARS-CoV-2 diversification in the United States: Establishing a pre-vaccination baseline

**DOI:** 10.1101/2021.06.01.21258185

**Published:** 2021-06-04

**Authors:** Adam A. Capoferri, Wei Shao, Jon Spindler, John M. Coffin, Jason W. Rausch, Mary F. Kearney

**Affiliations:** 1 HIV Dynamics and Replication Program, National Cancer Institute, Frederick, Maryland, USA.; 2 Department of Microbiology and Immunology, Georgetown University, Washington D.C., USA.; 3 Advanced Biomedical Computing Science, Frederick National Laboratory for Cancer Research (FNLCR) sponsored by the National Cancer Institute, Frederick, Maryland, USA.; 4 Department of Molecular Biology and Microbiology, Tufts University, Boston, Massachusetts, USA.

## Abstract

In 2020, SARS-CoV-2 spread across the United States (U.S.) in three phases distinguished by peaks in the numbers of infections and shifting geographical distribution. We investigated the viral genetic diversity in each phase using sequences publicly available prior to December 15^th^, 2020, when vaccination was initiated in the U.S. In Phase 1 (winter/spring), sequences were already dominated by the D614G Spike mutation and by Phase 3 (fall), genetic diversity of the viral population had tripled and at least 54 new amino acid changes had emerged at frequencies above 5%, several of which were within known antibody epitopes. These findings highlight the need to track the evolution of SARS-CoV-2 variants in the U.S. to ensure continued efficacy of vaccines and antiviral treatments.

## One Sentence Summary

SARS-CoV-2 genetic diversity in the U.S. increased 3-fold in 2020 and 54 emergent nonsynonymous mutations were detected.

The first reported case of COVID-19 in the U.S. was on January 20^th^, 2020 (1). Since that time, through May 2021, there have been more than 32.9 million U.S. cases (20.2% of global) and 585,700 deaths (17.4% of global) (2). Several vaccines have been developed, tested, approved, and are now being administered in the U.S. and elsewhere. Although vaccine administration began less than a year after the first reported case in the U.S., expansive global spread of SARS-CoV-2 over this period allowed for the concomitant emergence of variants with greater transmissibility and virulence as well as partial resistance to current preventives and treatments (3, 4). Though they share some common genetic features, such ‘Variants of Concern’ (VOCs) appear to have emerged independently in different regions throughout the world, raising the question of whether, and how quickly, variants resistant to induced immunity will evolve in the face of new selection pressures imposed by widespread vaccine administration (5, 6).

**Fig. 1.**
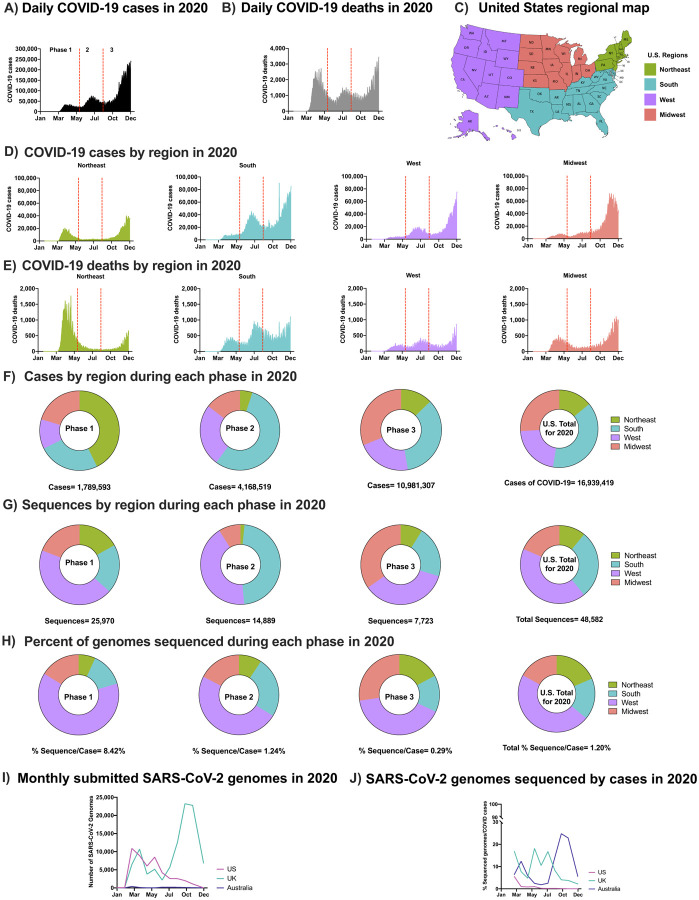
SARS-CoV-2 Epidemic in the U.S. in 2020 (**A**) Daily COVID-19 cases in the U.S. in 2020 (**B**) Daily COVID-19 deaths in the U.S. in 2020 (**C**) U.S. regional map colored by region (**D**) Number of COVID-19 cases in the U.S. in 2020 by region: Northeast, South, West, Midwest, respectively. (**E**) Number of COVID-19 deaths in the U.S. in 2020 by region. (**A-B & D-E**) Separation of Phases is denoted by vertical dotted red lines. Data were smoothed by a moving 3-day average. (**F**) Proportion of COVID-19 cases by region during each phase and the overall contribution to the U.S. total in 2020. (**G**) Proportion of SARS-CoV-2 sequences accessed (submission as of December 15^th^, 2020) by region during each phase and the overall contribution to the U.S. total in 2020 (**H**) The number of sequneces per case were obtained by each region during each phase and the U.S. total in 2020. (**F-H**) Highlights Phase 1, 2, and 3, followed with U.S. total of 2020. (**I**) Total number of sequences submitted to GISAID from the U.K., Australia, and the U.S. by December 15^th^, 2020. (**J**) Submitted SARS-CoV-2 genomes normalized to the number of COVID-19 cases from the U.K., Australia, and the U.S. (see Supplementary Methods).

The spread of SARS-CoV-2 in the U.S. in 2020 occurred in three phases marked by peaks in case numbers, hospitalizations, and deaths (Fig. 1A–B). Phase 1, in the winter and spring of 2020, began with the introduction of SARS-CoV-2 from Europe and Asia (7) and was followed by a surge of cases resulting from community spread and mobility (8) especially in the Northeast (9–12) (Fig. 1C–E). Phase 2 began in early June with accelerated community spread primarily in the South and West after mitigation policies were relaxed (Fig. 1D–E). The start of Phase 3 in the fall of 2020 was marked by a surge of transmission in the Midwest (Fig. 1D–E) followed by a nationwide increase, at or near the end of which time public vaccination was initiated (Fig. S1 & Table S1).

The regional distribution of COVID-19 cases varied by phase and was not always correlated with the level of viral sequencing in the different regions. For example, although the South region had the greatest overall number of cases (Fig. 1F), the majority of SARS-CoV-2 sequences were obtained from samples collected in the West (Fig. 1G–H). In total, viral sequences were obtained from 1.2% of reported U.S. cases in 2020, compared to 8.1% in the U.K. and 6.2% in Australia (Fig. 1I–J). The aggregate rate of sequencing in the U.S. reflects a decrease from 8.4% in Phase 1 to 0.3% in Phase 3, a difference that can be partly explained by the long intervals between sample collection and sequence deposition in GISAID (median: ~100 days; Fig. 1H–I). Of note, the rate of sequencing has significantly increased in 2021 compared to 2020.

To characterize the increasing genetic diversity and divergence of SARS-CoV-2 in the U.S. prior to both the introduction of vaccines and detection of the first VOC, high-coverage full-length genome sequences with GISAID submission dates on or before December 15^th^, 2020 were analyzed. All early GISAID-assigned clades of SARS-CoV-2 (G/GH/GR/S/L/V) were identified in the U.S. in Phase 1. However, the G-based clades (G/GH/GR), defined by the D614G mutation in the Spike (S) gene (13, 14), accounted for >99% of sequences by Phase 2 (Fig. S2). SARS-CoV-2 variants with the D614G mutation have been shown to be more infectious and exhibit some degree of resistance to certain monoclonal antibodies (15), yet they maintain convalescent serum neutralization sensitivity (16) and do not appear to worsen clinical outcomes (17).

**Fig. 2.**
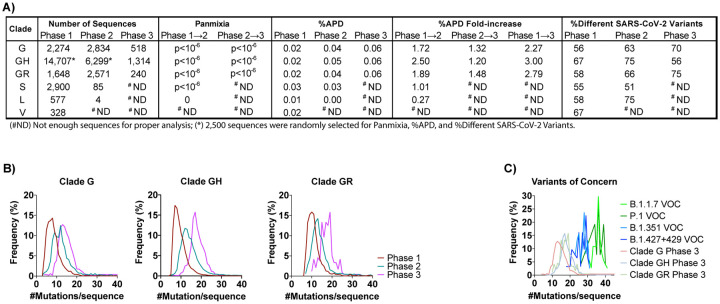
SARS-CoV-2 genetic diversity increased over time. (**A**) The number of sequences obtained from GISAID (https://www.gisaid.org/) in the analysis for each clade by Phase are reported. Genetic divergence was measured by panmixia probability (18) (significance cutoff, p<10^−3^) for clade/Phase with >11 sequences for Phase 1→2 and Phase 2→3. Genetic diversity was measured by average pair-wise distance (%APD) and the percent of different SARS-CoV-2 variants. (**B**) The distribution of the frequency for the number of mutations per sequence relative to the Wuhan-Hu-1 isolate was determined for the G-based clades in each Phase. (**C**) Distribution of the frequency for the number of mutations per sequence for the VOCs: B.1.1.7 (20I/501Y.V1, GR, first observed in U.K.), P.1 (20J/501Y.V3, GR, Brazil), B.1.351 (20H/501Y.V2, GH, South Africa), and the collective B.1.427/429 (20C/S:452R, GH, U.S.-California). (^#^ND) Not enough sequences for proper analysis; (*) 2,500 sequences were randomly selected for Panmixia, %APD, and %Different SARS-CoV-2 Variants.

The aggregate average pair-wise distance (APD) among G-based clades increased from 0.02% in Phase 1 to 0.06% in Phase 3, reflective of 2.3-, 3.0-, and 2.8-fold increases for clades G, GH, and GR, respectively (Fig. 2A & Fig. S3). Additionally, the approximate rate of change in APD in the G clades was 1.95-nt/month (clade G), 2.85-nt/month (clade GH), and 2.22-nt/month (clade GR) (Fig. S3A). In contrast, the APD of sequences comprising the VOCs (B.1.1.7, P.1, B.1.351, and B.1.427/429) was between 0.02% and 0.06%, and were within the interquartile range of the diversity measured in the G clades in the U.S. in 2020 despite being introduced towards 2021 (Fig. S3B). The observed increase in genetic diversity of SARS-CoV-2 in the U.S. in 2020 was due to an increase in the number of unique variants comprising clade G (+14%) and clade GR (+17%) (Fig. 2A). However, despite the 3-fold increase in APD, clade GH had an overall decrease in the number of different variants from Phase 1 to Phase 3 (−11%) (Fig. 2A). This finding suggests that the increase in genetic diversity in the GH clade resulted not from an increase in the number of variants but rather from the spread of fewer variants that were more highly divergent. This finding is further supported by observations of the numbers of mutations observed per sequence over time for each clade (Fig. 2B). Specifically, whereas in Phase 1 clades G, GH, and GR averaged 7, 7, and 10 mutations/sequence, respectively, these frequencies increased by 1.7-, 2.4-, and 1.8-fold in Phase 3, another indication of a disproportionate increase in GH clade divergence. For comparison, the VOCs had 26–36 mutations/sequence compared to their clade consensus (Fig. 2B–C). Similarly, panmixia, a metric indicating the degree to which random populations remain unstructured (*i.e.*, non-divergent) over time (18), was calculated for the respective clades. We found there was significant divergence in both the G- and S-based clades from Phase 1 to Phase 2 (*p<10*^*−6*^), demonstrating there was not random unstructured population mixing and that viral evolution was directional (Fig. 2A).

Levels of SARS-CoV-2 sequencing in the U.S. determine both the sensitivity with which we are able to detect emerging variants and our degree of statistical confidence that we have done or can do so. From U.S. viral sequences submitted to GISAID prior to Dec 15, 2020, we were able to detect mutations present in the population at frequencies ≥5% in Phases 1 and 2 and ≥14.5% in Phase 3 with 95% confidence (see Supplementary Methods). Increasing the sensitivity of and confidence in this type of analysis in 2021 will require greater sampling as is now being pursued. For instance, to detect mutations present in each daily sample at 1% or 0.1% frequency with 99% confidence, the viral sequencing capacity in the U.S. needs to increase by ~7.8-fold (to 460 sequences/day) or ~78-fold (4,600 sequences/day), respectively (Fig S4), a goal that is now within reach due to increases in funding for SARS-CoV-2 sequencing. For this study, majority-rule consensus sequences of each G clade from Phase 1 were compared to all respective sequences in the subsequent two phases (Table S2–4). Only mutations present in ≥5% of the population were included in the analyses, although mutations of specific interest present at lower frequencies were also noted and are discussed below.

**Fig. 3.**
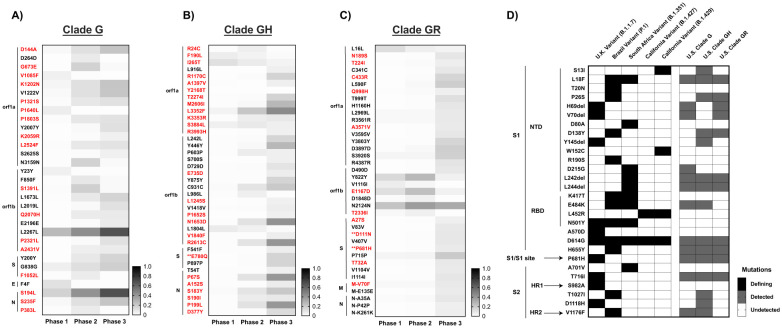
Emerged mutations in SARS-CoV-2 have increased in the U.S. (**A-C**) Non-clade defining mutations of Clades G, GH, and GR. Emerging mutations are shown in which, during at least one Phase in 2020 the frequency exceeded 5%. Sequences were compared to the majority-consensus sequence for each respective clade in Phase 1. Mutation designations reflect the relative amino acid positions in the gene regions. There were no common mutations among the G-based clades. Red text denotes non-synonymous mutations. (**) Mutations that occur in T- or B-cell epitope regions. (**D**) Comparison of non-synonymous mutations and deletions in Spike between VOCs and the U.S. G-based clades across all three phases. The presence or absence of mutations is indicated by shading: VOC-defining mutations (black), mutations detected during at least one phase in the respective G-clade (gray), and undetected in either VOC or U.S. G-clades (white).

About half of the new mutations that arose in the U.S. and persisted at frequencies ≥5% were nonsynonymous, *i.e.*, clade G: ORF1a (9 nonsynonymous mutations), ORF1b (4), Spike (1), Nucleocapsid (3); clade GH: ORF1a (12), ORF1b (6), Spike (1), Nucleocapsid (6); and clade GR: ORF1a (5), ORF1b (2), Spike (4), Matrix (1). While some mutations arose and then declined during 2020 (or shifted geographically), most persisted and increased in frequency (Fig. 3A–C). In particular, clade G mutation N^S194L^ and clade GH ORF1a^L3352F^, ORF1b^N1653D;R2613C^ increased more than 40% from Phase 1 to Phase 3. Many new mutations were detected in Phase 3 despite the limitations of extremely shallowing sampling.

Since all current vaccines were designed to potentiate cellular and humoral immune memory responses against the SARS-CoV-2 Spike protein (19), the emergence of mutations in the viral gene encoding Spike warrant special attention. In accordance with this elevated level of importance, VOCs are defined primarily, though not exclusively, by their associated Spike mutations. Critical investigations have been and are being conducted to determine how emergent Spike mutations affect vaccine efficacy (20, 21), as well as effectiveness of immunotherapeutics (*i.e.*, monoclonal antibodies and convalescent plasma) (3, 21), and viral transmission and pathogenesis (13, 17, 22). Selection of Spike mutations among recipients of convalescent plasma is particularly concerning (23–25), since the partial protection conferred by such treatments is likely conducive to immune escape, and may have contributed to the emergence of the B.1.1.7, B.1.351, P.1, and B.1.427/429 VOC (26).

Although the VOCs emerged later in 2020, our analysis shows that many of their defining mutations were present individually in the U.S. at low frequency (<1% of total sequences) as early as Phase 1 (Fig. 3D). However, with the exception of S^P681H^, the prevalence of these mutations never reached 5% in any of the 3 Phases, and they were therefore excluded from our primary analysis. Mutations defining the B.1.427/429 VOC (S^S13I;W152C;L452R^) which originated in the U.S., were only detected in a very small fraction of sequences in Dec 2020, possibily due to the lag between sample collection and sequence deposition and/or shallow Phase 3 sampling. The S^E484K^ mutation has been identified in multiple VOCs and is particularly concerning, having also been reported to contribute to immune evasion (3). At this time, however, immunity induced by mRNA-based vaccines appears to remain at least partially effective against S^E484K^ – containing VOCs (27–30).

Although the replication fidelity of SARS-CoV-2 is quite high relative to other RNA viruses (5, 31), its genetic stability is countered by the expansive spread of the virus both in the U.S. and globally. On balance, evolution of this virus might be best characterized as slow but inexorable, driven largely by genetic drift but also influenced by selective pressures such as relative infectivity, relative transmissibility, and perhaps even immune evasion. Conversely, the relatively recent and suddenly emergent VOCs are characterized by a significantly higher degree of genetic divergence that is rapidly acquired and clearly confers a replicative advantage. These variants were most likely the product of isolated cases in which the evolutionary environments differ substantially from the norm; *e.g.*, from chronically infected immunosuppressed individuals including those who received treatment with monoclonal antibodies or convalescent serum (23, 25, 32).

Regardless of the evolutionary pathways by which they emerge, the emergence of mutations in both the U.S. and global viral populations threatens the continued efficacy of current treatments and vaccines and will do so increasingly as the virus continues to spread and evolve. In response, scientists worldwide are coming together to increase timely SARS-CoV-2 sequencing and analysis to help guide decisions on current and future health policies. Unfortunately, in 2020, efforts to slow the spread of the virus within the U.S. was hampered by low adherence to recommended preventative measures (*e.g.*, mask wearing, hand washing, social distancing) as well as inconsistent and even conflicting messaging regarding these measures. These failures have indirectly accelerated viral divergence, increased genetic diversity, and resulted in the accumulation of nonsynonymous mutations, many of which are within epitopes associated with resistance to neutralizing antibodies that may ultimately contribute to immune evasion.

As more people are vaccinated, selective pressure for immune-resistant variants will increase concomitantly. As such, monitoring individuals that become infected with SARS-CoV-2 after vaccination, and those with prolonged infection (*e.g.*, immunocompromised individuals) is essential to understanding the capacity of SARS-CoV-2 for escape from vaccine-induced immunity. While our study focused on the U.S., it is important to recognize that the emergence of potential new VOCs is an ongoing and global concern. Therefore, we must maximize our vigilance for detecting and analyzing new variants, and just as importantly, continue to encourage measures to prevent their spread.

## Supplementary Material

1
