# Supplementary material for: 2020 SARS-CoV-2 diversification in the United States: Establishing a pre-vaccination baseline
Source: medRxiv. 2021 Jun 4:2021.06.01.21258185. Preprint. [Version 1] doi: 10.1101/2021.06.01.21258185 (PMC8202437; doi:10.1101/2021.06.01.21258185)
Supplement: 1 [file NIHPP2021.06.01.21258185V1-supplement-1.pdf]

medRxiv preprint doi: https://doi.org/10.1101/2021.06.01.21258185; this version posted June 4, 2021. The copyright holder for this preprint (which was not certified by peer review) is the author/funder, who has granted medRxiv a license to display the preprint in perpetuity. This article is a US Government work. It is not subject to copyright under 17 USC 105 and is also made available for use under a CC0 license.

Supplementary Materials for

2020 SARS-CoV-2 diversification in the United States: Establishing a pre-vaccination baseline

Adam A. Capoferri, Wei Shao, Jon Spindler, John M. Coffin, Jason W. Rausch, Mary F. Kearney
Correspondence to: adam.capoferri@nih.gov

This PDF file includes:

Materials and Methods
Figs. S1 to S5
Tables S1 to S5
References 32-44

medRxiv preprint doi: https://doi.org/10.1101/2021.06.01.21258185; this version posted June 4, 2021. The copyright holder for this preprint (which was not certified by peer review) is the author/funder, who has granted medRxiv a license to display the preprint in perpetuity. This article is a US Government work. It is not subject to copyright under 17 USC 105 and is also made available for use under a CC0 license.

Materials and Methods

COVID-19 Cases and Deaths

Data for U.S. COVID-19 cases and deaths were extracted from https://covidtracking.com/data from 01-January-2020 through 15-December-2020 (accessed 18-December-2020). U.S. regions were assigned based on the four Census Regions of the United States (U.S. Census Bureau) (*32*). The pandemic in the U.S. was divided into three phases based on COVID-19 case peaks where the derivative of the trough was approximately zero: Phase 1 of winter-spring (01-January-2020—31-Mary-2020), Phase 2 of summer (01-June-2020—31-August-2020), and Phase 3 of Fall (01-September-2020—15-December-2020). The estimated 2019 population for each U.S. State was accessed by the U.S. Census Bureau (Table S1) in order to normalize the incidence of COVID-19 cases and deaths in the sub-regional areas of the U.S. GraphPad Prism V.8.4.3 was used to visualize the data.

SARS-COV-2 Sequences

A total of 36,299 full-length (29,782 bp), high-coverage SARS-CoV-2 genomes from humans in the U.S. with infections between 20-January-2020 through 15-December-2020 were obtained from gisaid.org (accessed 18-December-2020) for sequence analysis. The numbers of sequences obtained were: Phase 1 (22,434), Phase 2 (11,893), and Phase 3 (2,072). Any nucleotide position with an 'N' was replaced with a gap. SARS-CoV-2 Clade O, Clade GV, Cruise-ship, and other U.S. territory sequences were excluded in the analysis due to the small representation. Variants of Concern (VOC or VOCs) from the United Kingdom (U.K.), South Africa, Brazil, and California were accessed from gisaid.org on 01 April 2021 (see also "Analysis of Variants of Concern" section of methods). The VOCs were sampled in the U.S. between 01 November 2020 through 31

medRxiv preprint doi: https://doi.org/10.1101/2021.06.01.21258185; this version posted June 4, 2021. The copyright holder for this preprint (which was not certified by peer review) is the author/funder, who has granted medRxiv a license to display the preprint in perpetuity. This article is a US Government work. It is not subject to copyright under 17 USC 105 and is also made available for use under a CC0 license.

March 2021. Fifty VOCs sequences were randomly selected for analyses and can be found in the **Supplemental File**. Sequences that contained 'Ns' were excluded. There was a total of 37 sequences (B.1.1.7 VOC), 38 sequences (B.1.351 VOC), 26 sequences (P.1 VOC), and 31 sequences (B.1.427/429 VOC) for the final dataset. Gap-stripped alignments were generated using the FFT-NS-1 200PAM/k=2 algorithm of MAFFT v7.450 (*33, 34*). Additional analyses of SARS-CoV-2 sequences were done using Geneious Prime® 2020.2.4 (*35*) and an in-house generated pipeline available at https://github.com/Wei-Shao/COV2-Analysis.

Distribution and genetic diversity of SARS-COV-2 in the U.S. in 2020

U.S. SARS-COV-2 genomes from each phase were separated by region and clade for each month in 2020. Monthly clade datasets with less than 10 sequences were excluded. To estimate the number of COVID-19 cases within each GISAID clade, the number of sequences was multiplied by the total monthly new COVID-19 cases. RStudio v1.3 (*36*) and GraphPad Prism V.8.4.3 were used to generate the figures.

The rate of SARS-COV-2 sequencing in the U.S. in 2020 was compared to the rate in the U.K. and in Australia. Sequence data for the U.K. and Australia was obtained under the same selection criteria as for the U.S. The number of cases were accessed on the same days using https://coronavirus.data.gov.uk/ and the National Notifiable Diseases Surveillance System (http://www9.health.gov.au/cda/source/rpt_3.cfm). The rate was determined from the number of sequences obtained monthly and the number of monthly cases of COVID-19 using in-house generated bioinformatic pipelines available at https://github.com/aacapoferri/COV2.

medRxiv preprint doi: https://doi.org/10.1101/2021.06.01.21258185; this version posted June 4, 2021. The copyright holder for this preprint (which was not certified by peer review) is the author/funder, who has granted medRxiv a license to display the preprint in perpetuity. This article is a US Government work. It is not subject to copyright under 17 USC 105 and is also made available for use under a CC0 license.

Genetic characterization and mutation analyses

Using in-house bioinformatic pipelines available at https://github.com/aacapoferri/COV2, SARS-COV-2 mutation frequencies were determined for each clade/Phase compared to either the majority-rule Phase 1 consensus sequence described next, or the Wuhan-Hu-1 reference genome (GenBank accession, NC_045512.2) or the VOCs (*37*). To exclude clade-defining mutations and amplification/sequencing errors, several steps were taken. First, majority-rule consensus sequences for each clade were generated from all genomes in Phase 1 and used as a reference in downstream analyses. This approach allowed majority clade-associated mutations to be omitted for the detection of new mutations only. Second, a threshold ≥5% frequency was used to eliminate mutations that were rarely detected and, therefore, could be PCR, sequencing errors, or real but not indicative of an emerging variant (see also "Number of sequences to detect mutations" section of methods). Mutation frequencies were plotted and annotated using the "Mutation frequency for SARS.R" script (an example provided on https://github.com/aacapoferri/COV2). Mutations that were above the ≥5% threshold were noted for each G-based clade and phase. The heatmaps for clades G, GH, and GR were generated to visualize the persistence and emergence of mutations present at frequencies ≥5% using GraphPad Prism V.8.4.3.

All sequences for each clade and Phase were included in each analysis with the exception of clade GH, where the number of sequences was too high for measurements of genetic diversity and divergence and, therefore, 2,500 sequences were randomly subsampled for those analyses. Mutation distributions were determined by assessing the number of mutations per sequence for each clade during each Phase. Statistical shifts in population structure (divergence) were determined using a test for panmixia with a statistical cut-off at $p<10^{-3}$ (*38*). Population genetic

medRxiv preprint doi: https://doi.org/10.1101/2021.06.01.21258185; this version posted June 4, 2021. The copyright holder for this preprint
(which was not certified by peer review) is the author/funder, who has granted medRxiv a license to display the preprint in perpetuity.
This article is a US Government work. It is not subject to copyright under 17 USC 105 and is also made available
for use under a CC0 license.

diversity was calculated as average pair-wise distance (APD) in MEGAX for each clade/Phase (*39*). These calculations were repeated with all clades during each month with at least 10 sequences or randomized sub-sampling of 50 sequences in triplicate to determine the APD. In cases where a particular clade had less than 10 sequences in a given month, they were excluded from analysis. Identical SARS-COV-2 genomes were collapsed to determine the number of different variants in the dataset. A simple linear regression was determined for clades G, GH, and GR in GraphPad Prism V.8.4.3 with the linear equation and goodness of fit R^2 reported. The slope was understood as the rate of change in %APD/month. The length of the SARS-CoV-2 genome is ~30,000 base pair, which when multiplied by the slope, gave an approximate number of nucleotide changes/month for a given G-based clade.

To examine the number of mutations per sequence in the G-based clades during each phase, the distribution of the number of mutations relative to the Wuhan-Hu-1 reference genome was plotted using in-house pipelines available at https://github.com/Wei-Shao/COV2-Analysis by Hamming distances.

Analysis of Variants of Concern (VOCs)

The four VOCs used in this study included the Pango lineage B.1.1.7 (Nexstrain 20I/501Y.V1, GISAID clade GR, originally isolated in the U.K.), P.1 (20J/501Y.V3, GR, Brazil), B.1.351 (20H/501Y.V2, GH, South Africa), and the B.1.427+429 (20C/S:452R, GH, California) sampled at locations in the U.S. After sequence sample processing, the number of mutations per sequence was determined for each VOC. The collection sampling on GISAID was set to any submitted sequences that spanned 5 months (November 2020-March 2021). We wanted to ensure that all

medRxiv preprint doi: https://doi.org/10.1101/2021.06.01.21258185; this version posted June 4, 2021. The copyright holder for this preprint (which was not certified by peer review) is the author/funder, who has granted medRxiv a license to display the preprint in perpetuity. This article is a US Government work. It is not subject to copyright under 17 USC 105 and is also made available for use under a CC0 license.

VOCs circulating in the U.S. would be captured. The individual VOC sequences collected for the final dataset in the U.S. were identified for 37 sequences of B.1.1.7 (February-March 2021), 26 sequences of P.1 (January-March 2021), 38 sequences of B.1.351 (January-March 2021), and 31 sequences of B.1.427+429 (December 2020 and January-February 2021).

The distribution of the number of mutations per sequence was compared to each respective derivative GISAID clade distribution during Phase 3, which was closest to the emergence of the VOC. The overall APD for each VOC dataset was calculated and compared to the genetic diversity of the G-based clades in the U.S. during 2020.

Due to the interest in the Spike protein of SARS-CoV-2 for vaccine and therapeutic strategies, VOC defining mutations in the S gene were specifically explored and were compared to sequences obtained from each Phase of infections in 2020. For this analysis mutations at frequencies less than 5% were included.

Potential effect of mutations

Majority-rule consensus sequences were generated for the G-based clades at each phase and were aligned to the Wuhan-Hu-1 reference genome. Previously mapped T-cell and B-cell epitopes from Spike and Nucleocapsid were annotated on the reference genome and included in Table S5 Nonsynonymous mutations observed in the G-based clades that differed from the reference in either T-cell or B-cell epitopes were noted.

Viral genetic surveillance resources

medRxiv preprint doi: https://doi.org/10.1101/2021.06.01.21258185; this version posted June 4, 2021. The copyright holder for this preprint (which was not certified by peer review) is the author/funder, who has granted medRxiv a license to display the preprint in perpetuity. This article is a US Government work. It is not subject to copyright under 17 USC 105 and is also made available for use under a CC0 license.

Several databases were used to compare and contrast global trends and our U.S.-based specific analysis. These included: PANGO lineages (https://cov-lineages.org/), NextStrain (https://nextstrain.org/sars-cov-2/), Global Initiative on Sharing Avian Influenza Data (https://www.gisaid.org/), Outbreak.info (https://outbreak.info/situation-reports), Coronavirus Resource Center (https://coronavirus.jhu.edu/map.html), Observable (https://observablehq.com/@spond/linkage-disequilibirum-in-sars-cov-2), Virological forum (https://virological.org/), Los Alamos National Laboratory (https://cov.lanl.gov/content/index), and the U.S. Centers for Disease Control and Prevention (https://www.cdc.gov/coronavirus/2019-ncov/cases-updates/variant-surveillance/variant-info.html).

medRxiv preprint doi: https://doi.org/10.1101/2021.06.01.21258185; this version posted June 4, 2021. The copyright holder for this preprint (which was not certified by peer review) is the author/funder, who has granted medRxiv a license to display the preprint in perpetuity. This article is a US Government work. It is not subject to copyright under 17 USC 105 and is also made available for use under a CC0 license.

Supplemental Figures and Tables

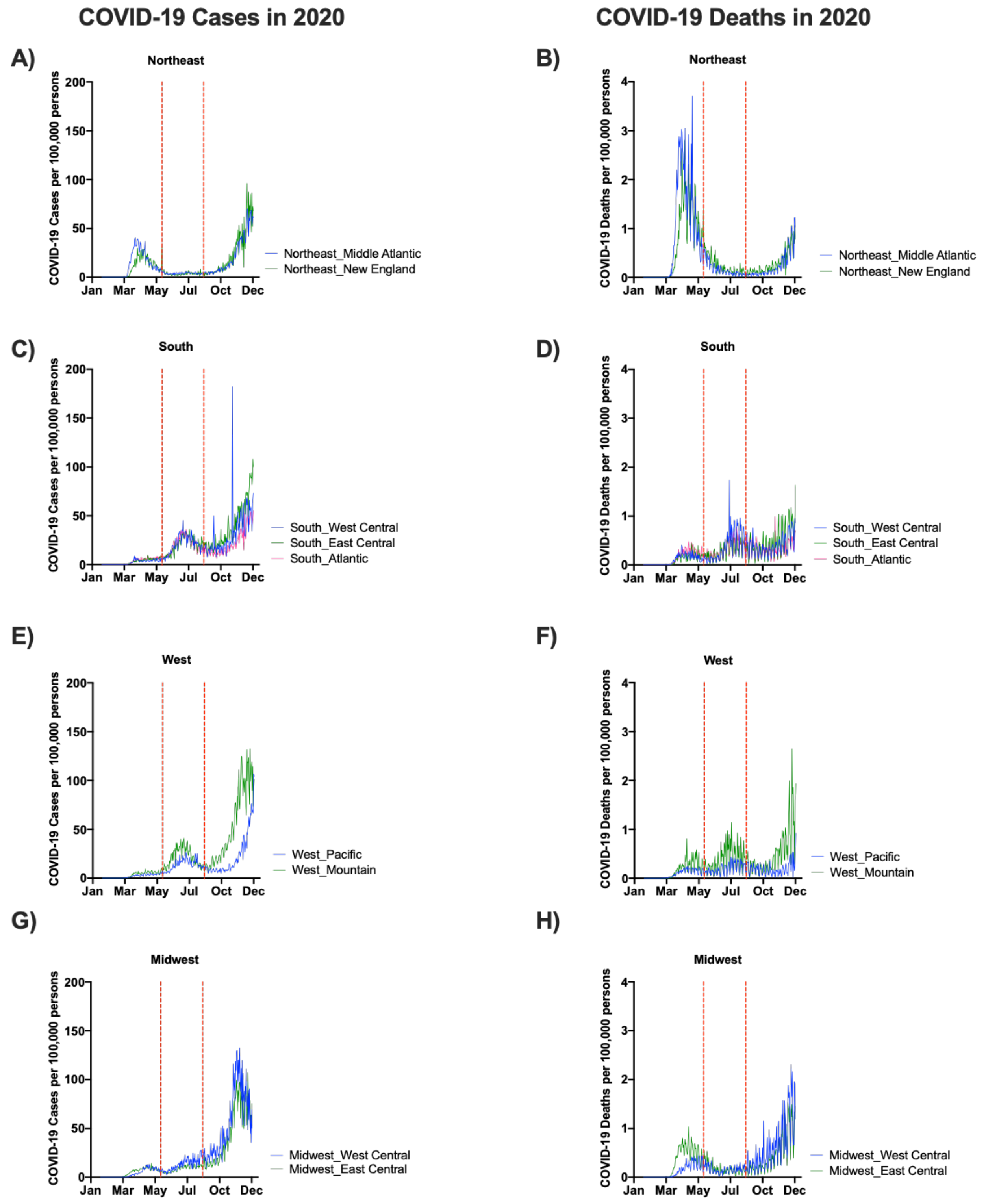

medRxiv preprint doi: https://doi.org/10.1101/2021.06.01.21258185; this version posted June 4, 2021. The copyright holder for this preprint (which was not certified by peer review) is the author/funder, who has granted medRxiv a license to display the preprint in perpetuity. This article is a US Government work. It is not subject to copyright under 17 USC 105 and is also made available for use under a CC0 license.

Fig. S1.
SARS-CoV-2 epidemic at the divisional level in the U.S. (**A, C, E, G**) Number of daily COVID-19 Cases in 2020 per 100,000 persons normalized to the 2019 estimated population in each sub-region. (**B, D, F, H**) Number of daily COVID-19 Deaths in 2020 per 100,000 persons normalized to the 2019 estimated population in each sub-region. (**A-B**) Northeast divisions of Middle Atlantic and New England. (**C-D**) Southern divisions of West Central, East Central, and Atlantic. (**E-F**) Western divisions of the Pacific and Mountain. (**G-H**) Midwestern divisions of West Central and East Central. Each division is colored respectively with dotted red lines indicating the separation of Phases. Population and date of first COVID-19 case in each sub-region is report in **Table S1**.

medRxiv preprint doi: https://doi.org/10.1101/2021.06.01.21258185; this version posted June 4, 2021. The copyright holder for this preprint (which was not certified by peer review) is the author/funder, who has granted medRxiv a license to display the preprint in perpetuity.
This article is a US Government work. It is not subject to copyright under 17 USC 105 and is also made available for use under a CC0 license.

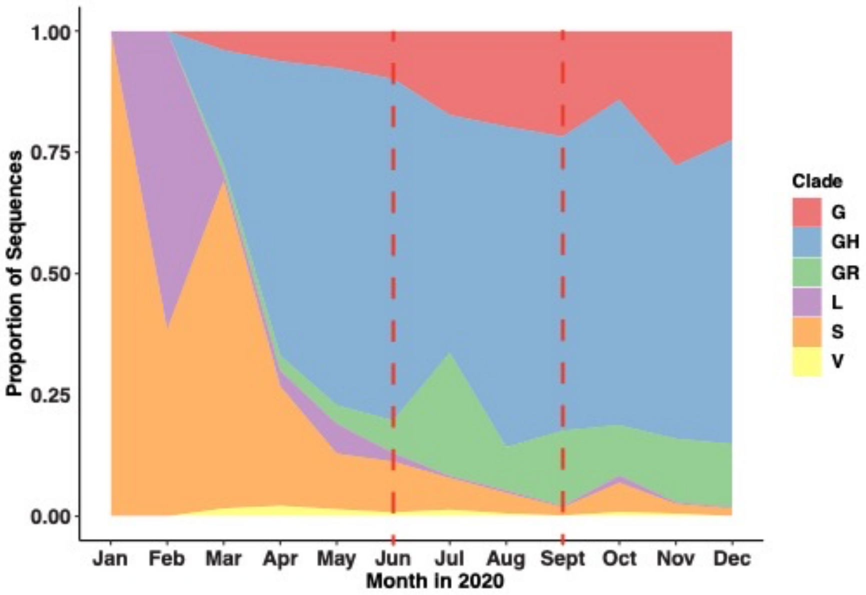

Fig. S2.

Proportion of SARS-CoV-2 genomes in each GISAID clade. The proportion of sequences observed during each month in 2020 based on the GISAID assigned Clades was calculated. The dotted red lines indicate the separation of Phases 1, 2, and 3. Data were accessed 18-December-2020 whereby any sequences submitted or collected by 15-December-2020 were considered. Clades G, GH, and GR represented the majority of all sequences by Phase 3.

medRxiv preprint doi: https://doi.org/10.1101/2021.06.01.21258185; this version posted June 4, 2021. The copyright holder for this preprint (which was not certified by peer review) is the author/funder, who has granted medRxiv a license to display the preprint in perpetuity. This article is a US Government work. It is not subject to copyright under 17 USC 105 and is also made available for use under a CC0 license.

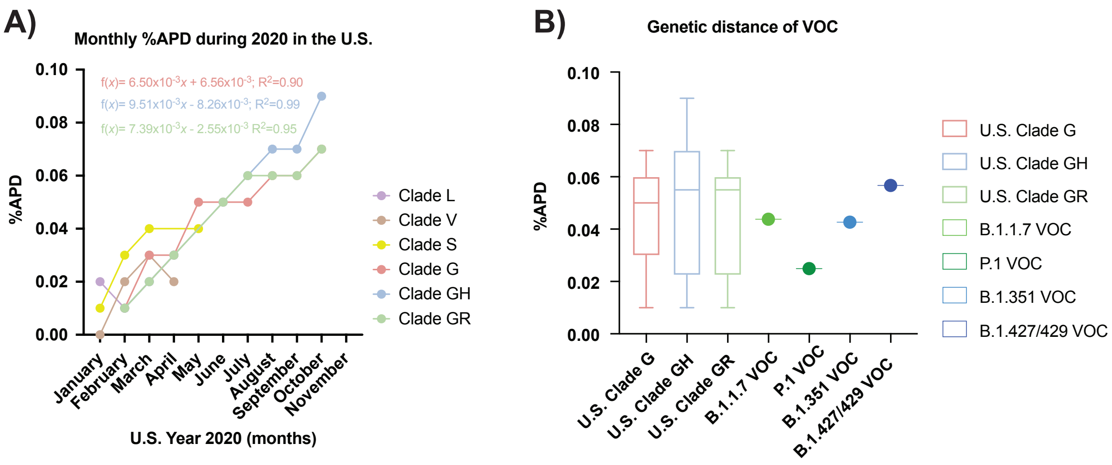

Fig. S3.

Genetic distance over time in 2020 for GISAID clades and Variants of Concern. (**A**) Average pairwise distance (APD) was calculated per month for sequences within each clade. Time points with less than 10 seqeuences were excluded. Three randomized subsamples of 50 sequences each were analyzed and the APD was calculated to ensure consistency between each subsampling. If there were 11-50 sequences for a clade at a given month, no subsampling was performed. A standard linear regression was run for clades G, GH, and GR. The rate of change in %APD over time is noted in the figure with the goodness of fit (R^2) reported. The %APD was plotted according to each respective month. The rate of change was 6.50×10^{-3} APD/month (clade G), 9.51×10^{-3} APD/month (clade GH), and 7.39×10^{-3} APD/month (clade GR); whch corresponded to 1.95 nt/month (clade G), 2.85 nt/month (clade GH), and 2.22 nt/month (clade GR) based on the SARS-CoV-2 genome of ~30,000 bp. (**B**) Comparison of the %APD between the VOCs and the G-based Clades. A box and whister plot of the %APD of the G-based clades in the U.S. during 2020 is shown. The median (solid line) is marked within the interquartile range, with the whiskers as the minimum and maximum. The VOC sequences sampled in the U.S. were accessed through GISAID.org with the collection date between 1 November 2020 to 31 March 2021. Total APD was calculated for the VOCs.

medRxiv preprint doi: https://doi.org/10.1101/2021.06.01.21258185; this version posted June 4, 2021. The copyright holder for this preprint (which was not certified by peer review) is the author/funder, who has granted medRxiv a license to display the preprint in perpetuity.
This article is a US Government work. It is not subject to copyright under 17 USC 105 and is also made available for use under a CC0 license.

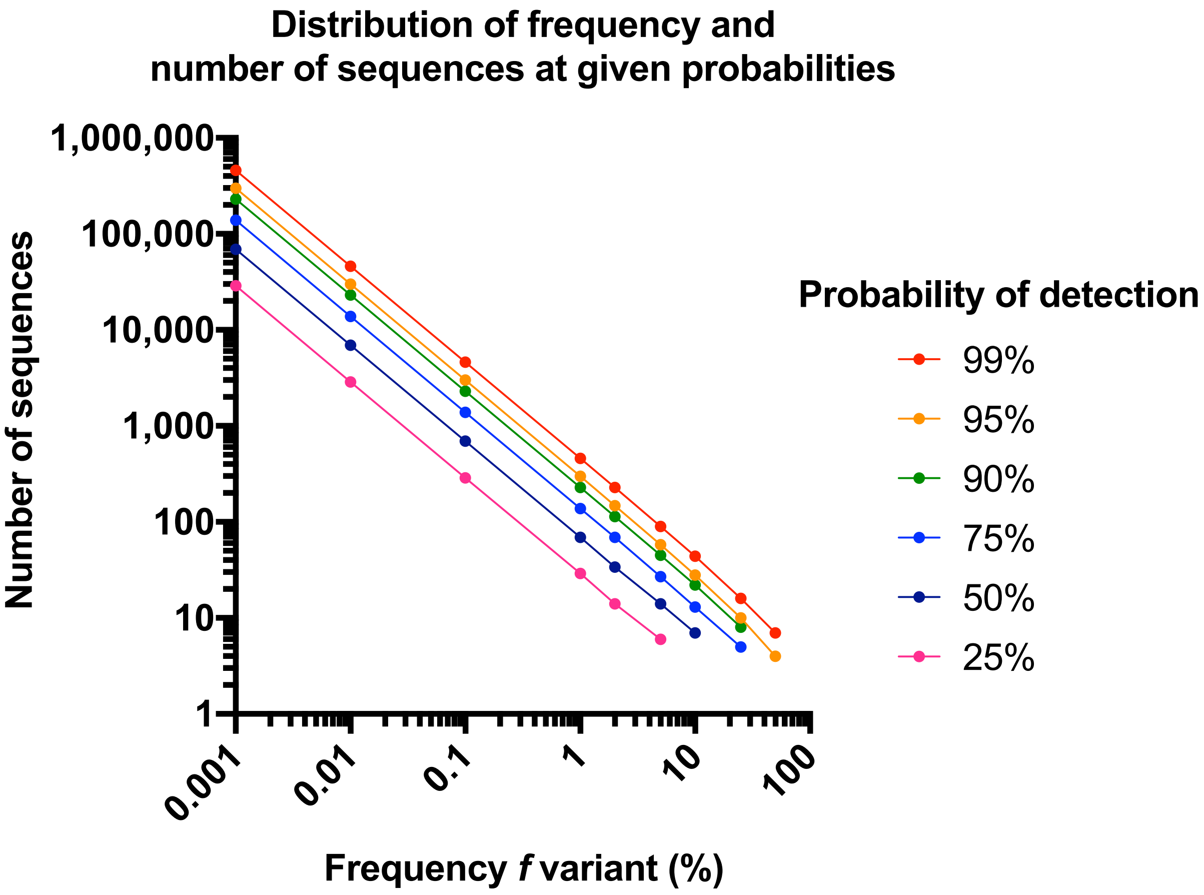

Fig. S4.
Determining number of sequences of sampling at a given probability to detect variant frequency. To determine the number of sequences required to detect at a certain mutation frequency with a given probability, follows the quotient: $N = \frac{\log(1-p)}{\log(1-f)}$. Where ($N$) is the number of sequences of any given sample that is required to be sampled to determine (f), the frequency the mutant detected, at a given (p), the probability of detecting the mutation at the provided frequency.

medRxiv preprint doi: https://doi.org/10.1101/2021.06.01.21258185; this version posted June 4, 2021. The copyright holder for this preprint (which was not certified by peer review) is the author/funder, who has granted medRxiv a license to display the preprint in perpetuity.
This article is a US Government work. It is not subject to copyright under 17 USC 105 and is also made available for use under a CC0 license.

Clade	Gene (amino acid position)	Phase	Epitope	U.S. Frequency	Description of epitope
G	N (221-235)		L L L L D R L N Q L E S K M **S**		T-cell MHC-II HLA-DR
		1	. . . . F	1.58%	
		2	. . . . F	11.29%	
		3	. . . . F	22.01%	
GH	S (776-785)		A L T G I A V E Q D K N T Q **E** F A Q V		IgG/IgA and B-cell antibody
		1	. . . .	0%	
		2	. . . . Q	0.04%	
		3	. . . . Q	15.98%	
GR	S (110-115)		L **D** S K T Q		B-cell antibody
		1	. . . . . .	0%	
		2	. N	0.12%	
		3	. N	8.75%	
GR	S (673-684)		Y Q T Q T N S S **P** R R A R		B-cell antibody
		1	. . . .	0%	
		2	. . . . H	0.12%	
		3	. . . . H	7.50%	

Fig. S5.

Mutations in G clades that are located in MHC-II HLA-DR T-cell and B-cell epitopes. Nucleocapsid MHC-I/II T-cell and Spike B-cell epitopes with detected mutations are shown. Nonsynonymous mutations were detected in Clades GH and GR starting in Phase 2 through Phase 3 (highlighted). The amino acid position of the epitope is denoted next to N (Nucleocapsid) and S (Spike). The U.S. frequency of detection is noted for each Phase. Epitopes examined are defined by **Table S5**.

medRxiv preprint doi: https://doi.org/10.1101/2021.06.01.21258185; this version posted June 4, 2021. The copyright holder for this preprint (which was not certified by peer review) is the author/funder, who has granted medRxiv a license to display the preprint in perpetuity. This article is a US Government work. It is not subject to copyright under 17 USC 105 and is also made available for use under a CC0 license.

Table S1

Demographics and Regional Division based on the U.S. Census Bureau. The 2019 estimated residential population for each State is reported. The first reported confirmed plus probable cases of COVID-19 are included (https://covidtracking.com/). The U.S. is generally divided into the Northeast, South, West, and Midwest. Where each major region can be divided into sub-regions. These estimated populations were then used to normalize the incidence of COVID-19 cases and deaths in the U.S. at the sub-regional level.

U.S. Region	U.S. Sub-region	State	Estimated Resident Population (2019)	Date of first reporting cases of COVID-19
Northeast (55,982,803)	Middle Atlantic (41,137,740)	NJ	8,882,190	05-March-2020
		NY	19,453,561	03-March-2020
		PA	12,801,989	06-March-2020
	New England (14,845,063)	CT	3,565,287	08-March-2020
		ME	1,344,212	12-March-2020
		MA	6,892,503	04-March-2020
		NH	1,359,711	04-March-2020
		RI	1,059,361	01-March-2020
		VT	623,989	08-March-2020
South (125,580,448)	West Central (40,619,450)	AR	3,017,804	12-March-2020
		LA	4,648,794	09-March-2020
		OK	3,956,971	07-March-2020
		TX	28,995,881	06-March-2020
	East Central (19,176,181)	AL	4,903,185	13-March-2020
		KY	4,467,673	07-March-2020
		MS	2,976,149	12-March-2020
		TN	6,829,174	05-March-2020
	Atlantic (65,784,817)	DE	973,764	11-March-2020
		FL	21,477,737	03-March-2020
		GA	10,617,423	04-March-2020
		MD	6,045,680	06-March-2020
		NC	10,488,084	04-March-2020
		SC	5,148,714	07-March-2020
		VA	8,535,519	08-March-2020
		D.C.	705,749	08-March-2020
		WV	1,792,147	18-March-2020
West (72,465,722)	Pacific (53,492,270)	AK	731,545	17-March-2020
		CA	39,512,223	04-March-2020
		HI	1,415,872	07-March-2020
		OR	4,217,737	05-March-2020
		WA	7,614,893	19-January-2020
	Mountain (18,973,452)	AZ	7,278,717	04-March-2020
		CO	5,758,736	04-March-2020
		ID	1,787,065	14-March-2020
		MT	1,068,778	12-March-2020
		NV	3,080,156	05-March-2020
		NM	2,096,829	12-March-2020
		UT	3,205,958	07-March-2020
		WY	578,759	12-March-2020
Midwest (66,682,283)	West Central (19,779,852)	IA	3,155,070	09-March-2020
		KS	2,913,314	08-March-2020
		MN	5,639,632	06-March-2020
		MO	6,137,428	08-March-2020
		NE	1,934,408	07-March-2020
		ND	762,062	12-March-2020
		SD	884,659	11-March-2020
	East Central (46,902,431)	IL	12,671,821	04-March-2020
		IN	6,732,219	06-March-2020
		MI	9,986,857	01-March-2020
		OH	11,689,100	09-March-2020
		WI	5,822,434	04-March-2020

medRxiv preprint doi: https://doi.org/10.1101/2021.06.01.21258185; this version posted June 4, 2021. The copyright holder for this preprint (which was not certified by peer review) is the author/funder, who has granted medRxiv a license to display the preprint in perpetuity. This article is a US Government work. It is not subject to copyright under 17 USC 105 and is also made available for use under a CC0 license.

Table S2.

Non-associated Clade G mutations that either persisted or emerged during 2020. Data were extracted where, during at least one Phase period, the frequency of a particular mutation was ≥5% compared to the clade Phase 1 majority consensus. The mutation specifies the nucleotide change as well as the amino acid change with coordinates of the gene and its product. Non-synonymous mutations are shown in red.

Gene	Mutation	Frequency (%)			Net Change (%)
		Phase 1	Phase 2	Phase 3	Phase 1→3
orf1a	a431c(D144A)	1.72	11.29	21.81	+20.09
	c792t(D264D)	0	7.59	0	0
	g2017a(G673E)	0	16.83	1.54	+1.54
	g3253t(V1085F)	5.1	0.07	0	-5.10
	g3606t(K1202N)	3.21	11.29	21.81	+18.60
	t3666c(V1222V)	1.54	11.04	21.62	+20.08
	c3961t(P1321S)	0.40	8.68	11	+10.60
	c4919t(P1640L)	6.02	0	0	-6.02
	c5407t(P1803S)	0.53	8.82	10.62	+10.09
	c6020t(Y2007Y)	0	1.24	11.20	+11.20
	a6176g(K2059R)	0	0.49	5.02	+5.02
	a7572c(L2524F)	0.40	8.57	10.23	+9.83
	c7875t(S2625S)	0.09	0.49	5.02	+4.93
	t9477c(N3159N)	0.13	17.29	1.54	+1.41
orf1b	c69t(Y23Y)	5.01	0.21	0.39	-4.62
	c2550t(F850F)	0.31	9.03	1.35	+1.04
	c4172t(S1391L)	0.40	18.38	1.54	+1.14
	c5019t(L1673L)	0.04	8.54	15.83	+15.79
	c6057t(L2019L)	0	1.2	10.81	+10.81
	g6210t(Q2070H)	0	6.99	7.53	+7.53
	a6588g(E2196E)	0	1.09	6.76	+6.76
	a6801g(L2267L)	25.99	43.9	67.37	+41.38
	c6962t(P2321L)	0	1.13	10.81	+10.81
	c7292t(A2431V)	0	5.75	7.14	+7.14
S	t600c(Y200Y)	0	8.57	15.83	+15.83
	t2514c(G838G)	5.5	12.14	18.15	+12.65
	c3156a(F1052L)	0.09	6.67	0	-0.09
E	c12t(F4F)	10.82	0.18	0	-10.82
N	c581t(S194L)	17.19	41.78	68.34	+51.15
	c704t(S235F)	1.58	11.29	22.01	+20.43
	c1148t(P383L)	0.13	6.81	0.19	+0.06

medRxiv preprint doi: https://doi.org/10.1101/2021.06.01.21258185; this version posted June 4, 2021. The copyright holder for this preprint (which was not certified by peer review) is the author/funder, who has granted medRxiv a license to display the preprint in perpetuity. This article is a US Government work. It is not subject to copyright under 17 USC 105 and is also made available for use under a CC0 license.

Table S3.

Non-associated Clade GH mutations that either persisted or emerged during 2020. Data were extracted where, during at least one Phase period, the frequency of a particular mutation was ≥5% compared to the clade Phase 1 majority consensus. The mutation specifies the nucleotide change as well as the amino acid change with coordinates of the gene and its product. Non-synonymous mutations are shown in red,

Gene	Mutation	Frequency (%) Phase 1	Phase 2	Phase 3	Net Change (%) Phase 1→3
orf1a	c70t(R24C)	0.05	5.35	0.23	+0.18
	t568c(F190L)	6.03	3.98	0.91	-5.12
	t794c(I265T)	11.29	7.40	4.19	-7.1
	g2748a(L916L)	0	0	5.18	+5.18
	c3508t(R1170C)	0	0.65	16.36	+16.36
	c4190t(A1397V)	0.03	0	5.18	+5.15
	c6821t(T2274I)	0.02	0.44	16.21	+16.19
	g7818a(M2606I)	0.03	2.16	27.09	+27.06
	c10054t(L3352F)	3.98	21.53	44.67	+40.69
	a10058g(K3353R)	0.19	6.10	8.60	+8.41
	c11651t(S3884L)	4.73	8.26	5.18	+0.45
	g11978a(R3993H)	0	0.02	5.10	+5.1
orf1b	t724c(L242L)	0.01	0.67	15.60	+15.59
	c1338t(Y446Y)	0.05	2.19	27.55	+27.5
	t1809a(P603P)	0.01	6.37	5.25	+5.24
	t2100c(S700S)	0	0	5.18	+5.18
	c2187t(D729D)	0.07	0.62	9.59	+9.52
	g2205t(E735D)	0.01	0.05	5.94	+5.93
	c2625t(Y875Y)	0.03	0.11	16.06	+16.03
	c2793t(C931C)	1.04	9.56	20.47	+19.43
	t2958g(L986L)	0.01	5.27	0.23	+0.22
	t3734c(L1245S)	0	0.02	5.02	+5.02
	g4254t(V1418V)	0.03	0.40	5.94	+5.91
	c4954t(P1652S)	0	0	5.02	+5.02
	a4957g(N1653D)	0.04	10.57	42.09	+42.05
	c5410t(L1804L)	6.78	4.38	0.99	-5.79
	g5518t(V1840F)	0.01	0.37	5.78	+5.77
	c7837t(R2613C)	0.06	10.02	40.64	+40.58
S	c1623t(F541F)	0.01	0.13	6.01	+6.00
	g2338c(E780Q)	0	0.41	15.98	+15.98
	a2691t(P897P)	0	0.64	16.06	+16.06
N	c162t(T54T)	0.01	0.06	5.18	+5.17
	c199t(P67S)	0.03	10.46	39.88	+39.85
	g454t(A152S)	0.01	0.08	6.32	+6.31
	c548a(S183Y)	1.03	9.53	20.70	+19.67
	g569t(S190I)	0.01	6.32	8.37	+8.36
	c596t(P199L)	0.04	10.26	39.80	+39.76
	g1129t(D377Y)	0.14	0.60	13.17	+13.03

medRxiv preprint doi: https://doi.org/10.1101/2021.06.01.21258185; this version posted June 4, 2021. The copyright holder for this preprint (which was not certified by peer review) is the author/funder, who has granted medRxiv a license to display the preprint in perpetuity. This article is a US Government work. It is not subject to copyright under 17 USC 105 and is also made available for use under a CC0 license.

Table S4.
Non-associated Clade GR mutations that either persisted or emerged during 2020. Data were extracted where, during at least one Phase period, the frequency of a particular mutation was ≥5% compared to the clade Phase 1 majority consensus. The mutation specifies the nucleotide change as well as the amino acid change with coordinates of the gene and its product. Non-synonymous mutations are shown in red.

Gene	Mutation	Frequency (%) Phase 1	Phase 2	Phase 3	Net Change (%) Phase 1→3
orf1a	c48t(L16L)	12.50	5.41	4.17	-8.33
	a566g(N189S)	0	0.04	9.17	+9.17
	c671t(T224I)	0.12	1.13	5.83	+5.71
	c1023t(C341C)	0	0.12	8.75	+8.75
	t1297c(C433R)	0	0.54	15.42	+15.42
	g1770t(L590F)	0	1.67	19.17	+19.17
	g2994t(Q998H)	0.30	1.05	5.42	+5.12
	a2997g(T999T)	0	0.08	11.67	+11.67
	t3480c(H1160H)	0	0.08	9.17	+9.17
	t8907c(L2969L)	2.25	1.59	7.92	+5.67
	a10683g(R3561R)	2.25	1.59	7.92	+5.67
	c10712t(A3571V)	0	0.12	7.50	+7.50
	c10785t(V3595V)	0	0.23	8.75	+8.75
	c11409t(Y3803Y)	0.12	0.27	15.42	+15.30
	c11691t(D3897D)	0.06	1.94	18.75	+18.69
	c11760t(S3920S)	3.40	3.31	17.92	+14.52
	c13161t(R4387R)	0	0.23	9.58	+9.58
orf1b	c1470t(D490D)	0	0.23	8.75	+8.75
	c2466t(Y822Y)	14.32	26.60	4.58	-9.74
	g3346a(V1116I)	6.13	2.92	0	-6.13
	g3501t(E1167D)	11.95	24.27	4.58	-7.37
	c5544t(D1848D)	0.91	5.02	0	-0.91
	t6372c(N2124N)	22.82	38.12	32.92	+10.1
	c7007t(T2336I)	2.06	5.10	0	-2.06
S	g79t(A27S)	0	0.35	5.42	+5.42
	c249t(V83V)	0	0	8.33	+8.33
	g331a(D111N)	0	0.12	8.75	+8.75
	c1221t(V407V)	0	0.12	8.75	+8.75
	c2042a(P681H)	0	0.12	7.50	+7.50
	c2145t(P715P)	3.46	3.62	20.00	+16.54
	a2194g(T732A)	0.18	0.74	7.92	+7.74
	a3312g(V1104V)	0	0.12	7.50	+7.50
	c3342t(I1114I)	0.06	0.31	9.17	+9.11
M	g208t(V70F)	2.31	1.63	7.92	+5.61
	a405g(E135E)	0	0.12	7.08	+7.08
N	g105t(A35A)	0.55	1.32	8.75	+8.20
	c126a(P42P)	0	0.04	5.42	+5.42
	a783g(K261K)	0	0.23	5.83	+5.83

medRxiv preprint doi: https://doi.org/10.1101/2021.06.01.21258185; this version posted June 4, 2021. The copyright holder for this preprint (which was not certified by peer review) is the author/funder, who has granted medRxiv a license to display the preprint in perpetuity.
This article is a US Government work. It is not subject to copyright under 17 USC 105 and is also made available for use under a CC0 license.

Table S5.

SARS-CoV-2 T-cell and B-cell epitopes in Spike and Nucleocapsid. Epitopes demonstrating cross-reactivity other human coronaviruses are denoted (#). In some studies, specific HLA alleles were examined for MHC-I/II. MHC-II HLA-DR allotypes included: DRB*01:01, DRB*03:01, DRB*04:01, DRB*07:01, DRB*11:01, and DRB*15:01 ($)

Epitope	Location	Restriction	Reference
MKDLSPRWYFYYLGTGPEAG	Nucleocapsid	MHC-II	(*40, 41*)
SKLWAQCVQLHNDIL	Nsp7	MHC-II	
HNDILLAKDTTEAFE	Nsp7	MHC-I	
MEVTPSGTWL	Nucleocapsid	MHC-I (HLA-B*40)	
LTDEMIAQY	Spike	MHC-I (HLA-A*01)	(*41*)
KTFPPTEPKK	Nucleocapsid	MHC-I (HLA-A*03)	
ATEGALNTPK	Nucleocapsid	MHC-I (HLA-A*03)	
QYIKWPWYI	Spike	MHC-I (HLA-A*24)	
KDGIIWVATEGALNT	Nucleocapsid	MHC-II (HLA-DR$#)	
GTWLTYTGAIKLDDK	Nucleocapsid	MHC-II (HLA-DR$#)	
RWYFYYLGTGPEAGL	Nucleocapsid	MHC-II (HLA-DR$#)	
ASWFTALTQHGKEDL	Nucleocapsid	MHC-II (HLA-DR$#)	
ASAFFGMSRIGMEVT	Nucleocapsid	MHC-II (HLA-DR$#)	
IGYYRRATRRIRGGD	Nucleocapsid	MHC-II (HLA-DR$#)	
LLLLDRLNQLESKMS	Nucleocapsid	MHC-II (HLA-DR$#)	
ITRFQTLLALHRSYL	Spike	MHC-II (HLA-DR$#)	
KYFKNHTSP	Spike	B-cell	(*42*)
TTKR	Spike	B-cell	
YYHKNNKSWM	Spike	B-cell	
ASTEK	Spike	B-cell	
AWNRKR	Spike	B-cell	
EQDKNTQ	Spike	B-cell	
GTNTSN	Spike	B-cell	
KYNENGT	Spike	B-cell	
LDSKTQ	Spike	B-cell	
PKKS	Spike	B-cell	
YQTQTNSPRRAR	Spike	B-cell	
VLTESNNKFLPFQQFGRDIA	Spike	B-cell	(*43*)
KPSKRSFIEDLLFNKVTLAD	Spike	B-cell	
ELDSFKEELDKYFKNHTSPD	Spike	B-cell	
SDSTGSNQNGER	Nucleocapsid	B-cell	(*44*)
TNSSPDD	Nucleocapsid	B-cell	
SRGGSQASSRSSSRSRNSSRNSTPGSSRGTS	Nucleocapsid	B-cell	
SGKGQQQQG	Nucleocapsid	B-cell	
KSAAESKKPPQKRT	Nucleocapsid	B-cell	
FPPTEPKKDKKKKADET	Nucleocapsid	B-cell	
GDGKMKDLSPRWYFYYLGTGPEAGLPYGANKDGIIWVATEG	Nucleocapsid	MHC-I	
RMAGNGGDAALALLLLDRLNQLES	Nucleocapsid	MHC-I	
KRTATKAYNVTQAFGRRGP	Nucleocapsid	MHC-I	
QFAPSASAFFGMSRIGMEVTPSGTWLTYTG	Nucleocapsid	MHC-I	
GGDGKMKDLSPRWYFYYLGTGPEAGLPYGANK	Nucleocapsid	MHC-II	
NGGDAALALLLLDRLNQLESKMSGKG	Nucleocapsid	MHC-II	
RQGTDYKHWPQIAQFAPSASAFFGMSRI	Nucleocapsid	MHC-II	

medRxiv preprint doi: https://doi.org/10.1101/2021.06.01.21258185; this version posted June 4, 2021. The copyright holder for this preprint (which was not certified by peer review) is the author/funder, who has granted medRxiv a license to display the preprint in perpetuity.
This article is a US Government work. It is not subject to copyright under 17 USC 105 and is also made available for use under a CC0 license.

References

32. in *The 2010 Census Regions and Divisions of the United States*. (2010).
33. K. Katoh, K. Misawa, K. Kuma, T. Miyata, MAFFT: a novel method for rapid multiple sequence alignment based on fast Fourier transform. *Nucleic Acids Res* **30**, 3059-3066 (2002).
34. K. Katoh, D. M. Standley, MAFFT multiple sequence alignment software version 7: improvements in performance and usability. *Mol Biol Evol* **30**, 772-780 (2013).
35. Geneious. vol. Prime 2020.2.4.
36. R. Team*,* P. RStudio, Ed. (2020).
37. F. Wu *et al.*, A new coronavirus associated with human respiratory disease in China. *Nature* **579**, 265-269 (2020).
38. M. R. Jordan *et al.*, Comparison of standard PCR/cloning to single genome sequencing for analysis of HIV-1 populations. *J Virol Methods* **168**, 114-120 (2010).
39. S. Kumar, G. Stecher, M. Li, C. Knyaz, K. Tamura, MEGA X: Molecular Evolutionary Genetics Analysis across Computing Platforms. *Mol Biol Evol* **35**, 1547-1549 (2018).
40. N. Le Bert *et al.*, SARS-CoV-2-specific T cell immunity in cases of COVID-19 and SARS, and uninfected controls. *Nature* **584**, 457-462 (2020).
41. A. Nelde *et al.*, SARS-CoV-2-derived peptides define heterologous and COVID-19-induced T cell recognition. *Nat Immunol* **22**, 74-85 (2021).
42. M. Zheng, L. Song, Novel antibody epitopes dominate the antigenicity of spike glycoprotein in SARS-CoV-2 compared to SARS-CoV. *Cellular & Molecular Immunology* **17**, 536-538 (2020).
43. E. Shrock *et al.*, Viral epitope profiling of COVID-19 patients reveals cross-reactivity and correlates of severity. *Science* **370**, (2020).
44. S. C. Oliveira, M. T. Q. de Magalhães, E. J. Homan, Immunoinformatic Analysis of SARS-CoV-2 Nucleocapsid Protein and Identification of COVID-19 Vaccine Targets. *Frontiers in Immunology* **11**, (2020).
